# Parvoviruses in Blood Donors and Transplant Patients, Italy

**DOI:** 10.3201/eid1401.070610

**Published:** 2008-01

**Authors:** Daniela Vallerini, Patrizia Barozzi, Chiara Quadrelli, Raffaella Bosco, Leonardo Potenza, Giovanni Riva, Gina Gregorini, Silvio Sandrini, Andrea Tironi, Giuliano Montagnani, Marisa De Palma, Giuseppe Torelli, Eric Delwart, Mario Luppi

**Affiliations:** * University of Modena and Reggio Emilia, Azienda Ospedaliera Policlinico, Modena, Italy; † University of Brescia, Spedali Civili, Brescia, Italy; ‡ Blood Systems Research Institute, San Francisco, California, USA; 1 These authors contributed equally to the study.

**Keywords:** blood donors, PARV4/5, transplantation, letter

**To the Editor:** Parvoviruses (PARV) 4 and 5 are 2 genotypes of a novel human parvovirus, with 92% nucleotide identity, identified in the plasma sample of a patient screened for acute HIV infection and in samples of manufactured plasma pools (*1*,*2*). Recently, PARV4 and PARV5 were identified in blood samples from 3 of 26 cadavers from the United Kingdom, all of whom were positive for hepatitis C virus RNA and had a history of intravenous drug use (*3*). PARV4/5 were also found in bone marrow (BM) and lymphoid tissues from 17 of 24 HIV-positive cadavers from Scotland (*4*) and in BM aspirates from 16 of 35 Italian patients with AIDS (*5*). Little or no information is available about the epidemiology and clinical correlates of infection with these novel viruses. To provide insights into their pathogenic potential in vivo, we assessed the frequency of PARV4/5 viremia in healthy patients, transplant patients, and those with suspected viral disease.

We performed a retrospective molecular study for the presence of PARV4/5 sequences in 4 groups of 417 Italian HIV-negative persons. Group 1 consisted of 100 blood donors recruited from the Transfusion Centre of Modena (northern Italy); group 2, 84 patients with hematologic diseases showing clinical signs of viral etiology but negative results for the most common viruses (herpesviruses, adenovirus, hepatitis virus, and coxsackie virus). For both of these groups, DNA was extracted for analysis from serum specimens and peripheral blood mononuclear cells (PBMCs). Groups 3 and 4 comprised recipients of kidney and allogeneic BM/peripheral blood stem cell (PBSC) transplants, for which DNA was extracted from serum specimens collected at 6 and 12 months, respectively, after transplantation. The nested PCR method was used to amplify a shared sequence of PARV4 and its variant PARV5 and was specific for the open reading frame 1. First step PCR was performed as previously described (*2*) with a sensitivity of 1–10 copies, on 1 μg PBMC DNA and on one fifth of DNA extracted from 0.25 mL of serum. Primers for second round PCR were PV4NS1Fn2 (5′-GTTGATGGYCCTGTGGTTAG-3′) and PV4NS1Rn2 (5′-CCTTTCATATTCAGTTCCTGTTCAC-3′). All positive results were confirmed by direct sequencing.

We found 3 positive case-patients, including 2 renal transplant recipients and 1 patient with a suspected viral disease; none of the blood donors tested positive on single-round PCR. On nested PCR, 1 blood donor had positive results; the positivity rate did not increase in the other groups (Table). In the first 2 groups, PARV4/5 sequences were detected only in the serum samples, not in the PBMCs collected at the same time. These sequences suggest that PBMCs are not a major site of viral replication. Similar to B19 infection, which is rarely reactivated in the setting of BM/PBSC transplantation (*6*,*7*), none of the BM/PBSC transplant patients were PARV4/5 positive. The detection of PARV4/5 sequences in the serum collected at 12 months after transplantation was not associated with the occurrence of any symptoms in the 2 renal recipients. Of note, the available serum samples collected from both recipients before transplantation, and at 6 and 24 months after transplantation, were PARV4/5 negative, which suggests that asymptomatic PARV4/5 infection may transiently occur after solid organ transplantation or may be acquired throughout transfusion or transplantation. Similarly, the rate of B19 infection in solid organ transplant recipients is low (1.4%–1.8%), and most B19 DNA–positive patients remain asymptomatic (*8*,*9*).

**Table:** Analysis of 417 patients tested for parvoviruses 4/5 by PCR*

Group	No. cases positive on serum samples/ no. tested (%)			No. cases positive on PBMCs/ no. tested	
	First-round PCR	Nested PCR		First-round PCR	Nested PCR
Blood donors	0/100	1/100 (1)		0/100	0/100
Patients with suspected viral diseases	1/84 (1.2)	1/84		0/84	0/84
BM/PBSC transplant recipients	0/107	0/107		ND	ND
Kidney transplant recipients (%)	2/126 (1.6)	2/126		ND	ND

*PBMCs, peripheral blood mononuclear cells; BM, bone marrow; PBSC, peripheral blood stem cell; ND, not done.

PARV4/5 sequences were detected in the serum collected from 1 patient affected with Wegener granulomatosis. This patient was under long-term treatment with steroids, concomitant with the development of a clinical syndrome for which a viral cause was suspected, including fever, severe anemia, a histologic-examination–proven postinfectious glomerulonephritis, and eryhtroid hypoplasia, with dsyserythropoiesis and dismegakaryopoiesis on BM examination. Serologic and molecular tests for the most common viruses, including B19, were negative and the patient died of multiple organ failure 1 month later. Single-cell PCR performed on the DNA extracted from isolated BM erythroid and myeloid progenitors in the formalin-fixed, paraffin-embedded BM tissue biopsy specimens, collected 2 days before death and at autopsy, were PARV4/5 negative. While the PARV4/5 viremia, in the absence of other known viral agents, suggests a possible contribution of this novel parvovirus to the patient’s clinical syndrome, the absence of the virus in the BM cells suggests that its in vivo tropism may markedly differ from that of B19.

In conclusion, although the frequency of PARV4/5 viremia is very low in the general Italian population, it is slightly higher in certain subgroups of iatrogenically immunosuppressed patients and it is not clear to which extent immunosuppression enhances viral reactivation and/or primary infection. Failure to detect PARV4/5 DNA in all but 4 study patients does not necessarily indicate a rarity of past viral exposure or infection in transplant patients or indeed in the general population. Further studies are needed to confirm a possible pathogenic role of PARV4/5 infection,
